# Effects of Dietary Thraustochytrid *Schizochytrium* sp. and Other Omega-3 Sources on Growth Performance, Carcass Characteristics, and Meat Quality of Broilers

**DOI:** 10.3390/ani12091166

**Published:** 2022-05-02

**Authors:** Jin-Joo Jeon, Hee-Jin Kim, Hwan-Ku Kang, Chan-Ho Kim, Hyun-Soo Kim, Eui-Chul Hong, Aera Jang, Sang-Ho Kim

**Affiliations:** 1 Poultry Research Institute, National Institute of Animal Science, Rural Development Administration, Pyeongchang 25342, Korea; khj0175@korea.kr (H.-J.K.); magic100@korea.kr (H.-K.K.); kindkims1@korea.kr (H.-S.K.); drhong@korea.kr (E.-C.H.); 2 Animal Welfare Research Team, National Institute of Animal Science, Rural Development Administration, Jeonju 55365, Korea; kch8059@korea.kr; 3 Department of Animal Life Science, Kangwon National University, Chuncheon 24341, Korea; ajang@kangwon.ac.kr; 4 K-AniWel Co., Ltd., Suwon 16672, Korea; kims2051@gmail.com

**Keywords:** broiler, DHA, omega-3 fatty acids, salmon oil, flaxseed oil, *schizochytrium*

## Abstract

**Simple Summary:**

Thraustochytrid *schizochytrium* is one of the promising microalgae for omega-3 production. We evaluated the possibility of *schizochytrium* as omega-3 source comparing with other omega-3 sources that have been used before. The main results were the increased levels of DHA in the thigh meat of broilers fed SP, while EPA and DHA were on the rise in the meat of broilers fed SO, and ALA was increased in the meat of broilers fed FO. The ω-6/ω-3 ratio of the thigh meat improved in all treatments, approaching the guideline levels without adverse effects on the productive performance, carcass traits, and thigh meat quality. Hence, our results suggest that salmon oil, flaxseed oil, and *schizochytrium* could be used as omega-3 fatty acids sources to improve the omega-3 level and ω-6/ω-3 balance in broiler thigh meat and to allow consumers to easily enhance their intake of these vital nutrients for health benefits.

**Abstract:**

Background: Looking for alternative omega-3 sources in broiler nutrition, microalgae began to get attention. We suspected that *schizochytrium* might play a similar role as other omega-3 sources that have been used before. Methods: 20 g/kg s*chizochytrium* powder (SP), salmon oil (SO), and flaxseed oil (FO) in each of the three treatment groups were supplemented in the basal diet (CON), and productive performance, carcass traits, and thigh meat quality of broilers were evaluated. Results: There was a significantly higher weight gain in the SP treatment compared to the other groups, but no difference was found in feed intake and feed conversion ratio. Thiobarbituric acid reactive substance values increased during storage in all the treatments but were significantly lower for SP than for SO and FO after 7 days of storage. Among the ω-3 fatty acids (FAs), α-linolenic acid increased the most in the FO treatment, eicosapentaenoic acid increased the most in the SO treatment, and docosahexaenoic acid increased the most in the SP treatment in thigh meat, reflecting the FA composition of the lipid source diets. Conclusions: We suggested that all the dietary ω-3 FA sources could improve the FA composition of chicken meat and our results indicated the possibility to supplement broiler diets with 2% level of SP, SO, and FO as ω-3 FA sources to produce meat with a good nutritional quality for consumer’s health benefits.

## 1. Introduction

The dietary consumption ratio of omega-6 to omega-3 (ω-6/ω-3) fatty acids (FAs) was close to 1:1 in the past 100–150 years before dietary patterns changed [1]. The intake of ω-3 polyunsaturated FAs (PUFAs) in humans, particularly docosahexaenoic acid (DHA, 22:6) and eicosapentaenoic acid (EPA, 20:5), has been declining with an increase in consumption of instant food, western diets, and meat from animals fed corn- and soybean-based diets, which are deficient in ω-3 FAs. Corn and soybean meal are the main components of poultry diets and enrich poultry products in ω-6 FAs. Consequently, the ω-6/ω-3 ratio increased to 15–16.7:1 in poultry products [2]. This higher level of ω-6 FAs and an increased ω-6/ω-3 ratio contribute to the pathogenesis of diseases, including cardiovascular disease, inflammation [2], and obesity [3]. The Minister of National Health and Welfare Canada [4] recommended an ω-6/ω-3 ratio of 4:1 in human diets. To increase the content of ω-3 FAs in animal muscles, dietary supplementation with lipids that are rich in ω-3 FAs has been used [5,6].

In trying to increase the ω-3 FA content in poultry products, the common practice is to add fish oil and flaxseed to a feed [7]. Generally, fish oils such as salmon oil (SO) are rich sources of DHA and EPA, and flaxseed oil (FO) is rich in α-linolenic acid (ALA). Marine algae, another ω-3 source, have also received increasing attention as a potential animal feed supplement because of their abundance of ω-3 FAs [8,9]. *Schizochytrium* sp., the marine algae utilized in the present study, is a rich source not only of ω-3 FAs (especially DHA) but also of antioxidants. *Schizochytrium* spp. Have been generally recognized as safe (GRAS) for use in chicken feed at levels up to 2.8% for broilers and 4.7% for layers [10]. Barclay et al. [11] reported that a product derived from *schizochytrium* had a golden hue, owing to naturally generated carotenoids, which may stabilize oxidation of ω-3 FAs [6]. *Schizochytrium* prevents lipid oxidation because it naturally produces a large amount of canthaxanthin (91.62 µg/g), as well as enzymes such as protease, lipase, and xylanase [12]. Currently, the effect of adding 2% of these omega-3 sources to the feed is unknown, and *schizochytrium* is also expected to have similar or better chicken meat quality improvement effects compared to previously used salmon oil and flaxseed oil.

Therefore, this study was performed to evaluate the effects of *schizochytrium*, SO, and FO as dietary sources of ω-3 FAs on the productive performance and carcass traits, as well as on the thigh meat quality and FA composition of broilers chickens. We also aimed to discover new ω-3 feed additives by comparing the effects of newly emerging marine microalgae with those of previously used additives as sources of ω-3 FAs.

## 2. Materials and Methods

All procedures used in present study were approved by the Animal Care and Welfare Committee of the National Institute of Animal Science, Rural Development Administration, Republic of Korea (NIAS2016-897).

### 2.1. Chickens and Experimental Design

A total of 480 male 1-day-old chickens (body weight (BW): 39.6 ± 0.40 g) of the commercial broiler strain Ross were raised for 35 days, which is the maximum slaughter age for broilers in Korea. All chicks were distributed in floor pens (width: 3.43 m; length: 2.56 m) in a room controlling the environment. We used a completely randomized design with four replicate pens, 30 chicks in each. A three-phase feeding program was used during the experiment, with a starter diet from 0 to 7 days, a grower diet from 8 to 21 days, and a finisher diet from 22 to 35 days (Table 1).

Within each stage, a basal diet was formulated to meet the NRC [13] for macro- and micronutrients for broiler chickens. The energy value of the feed mixture was calculated from the chemical composition based on the EU Regulation (EC 152/2009 annex VII): ME (MJ/kg) = (0.1551 × % crude protein + 0.3441 × % crude fat + 0.1301 × % total sugar + 0.1669 × % starch)/1000. Three dietary treatments were prepared by adding 20 g/kg SP, 20 g/kg SO, and 20 g/kg FO to the basal diet, respectively. The diets were in a mashed form. The temperature and humidity regimens were maintained in accordance with the recommendations of the Korea poultry feeding standards, and light was provided for 23–24 h from day 0 to day 7 and then 1L:2D until the end of the trials. Body weight gain (BWG) and feed intake (FI) were recorded over the 35-day experimental period. The feed conversion ratio (FCR) was measured as FI divided by BWG.

### 2.2. Preparation of Schizochytrium and Fatty acid Contents in Feed

*Schizochytrium* powder (SP) was obtained from the Jeonbuk National University (Iksan, Jeollabuk-do, Korea). *Schizochytrium* was cultured in a medium containing glucose, yeast extract, bay salt, and KH_2_PO_4_ and then dried into powder. Table 2 shows that the cultured *schizochytrium* contained 29.4% DHA and 63.4% palmitic acid and Table 3 shows *fatty* acid contents in diets with the addition of *schizochytrium powder* and *other omega-3 sources*.

### 2.3. Sampling Procedures

BWG and FCR were calculated to evaluate performance. We measured the body mass of all chicks at hatching and at 35 days of age. At the end of the experiment, five birds from each treatment group were randomly selected and slaughtered (cervical dislocation, bleeding, scalding, plucking, chilling, and dripping) for carcass evaluation after a 12 h starvation period. The dressing percentage was calculated by the weight of the commercial carcass (after removal of the viscera, neck, head, and shank) in proportion to the broiler live weight, and the abdominal fat pad (including fat surrounding the gizzard, bursa of fabricius, cloaca, and adjacent muscles) was removed and weighed. Five broilers were selected from each treatment to analyze thigh meat (*Peroneus longus*) qualities such as the water-holding capacity (WHC), pH, thiobarbituric acid reactive substances (TBARS), and the FA composition.

### 2.4. Proximate Analysis

Raw thigh meat was analyzed for dry matter (AOAC, 1990; method 934.01), crude protein (AOAC, 1990; method 988.05), crude fat (AOAC, 1990; method 920.39), and ash (AOAC, 1990; method 942.05) [14].

### 2.5. FA Composition of Thigh Meat with Skin

Lipid was extracted using a 2:1 chloroform/methanol mixture, based on the procedures by Folch et al. [15] and hydrolysis was performed according to Morrison and Smith [16]. The FA composition was determined by gas chromatography (Agilent 6890N; Agilent Technologies, Santa Clara, CA, USA) using a CP-Sil 88 capillary column (Agilent CP7489, 100 mm × 0.25 mm × 0.20 μm) and the following chromatographic conditions: injector temperature, 260 ℃ and helium as the carrier gas. The results were calculated as a percentage of the total peak area.

### 2.6. TBARS

Susceptibility of meat to lipid oxidation during storage at 4 °C was estimated at 1, 3, 5, and 7 days of storage by measuring TBARS values based on the procedures by Witte et al. [17]. Approximately 10 g of thigh meat was added to 25 mL of 20% trichloroacetic acid (in 2 M phosphoric acid) and homogenized (PolyTron PT-2500E; Kinematica, Lucerne, Switzerland). Then, 35 mL of the homogenate was diluted with 15 mL of distilled water and centrifuged at 4 °C (10 min at 3000 rpm). Five milliliters of the filtered supernatant were mixed with 5 mL of 0.005 mM 2-thiobarbituric acid and maintained at room temperature for 15 h, after which the malondialdehyde (MDA) content per kilogram of meat was obtained by measuring absorbance at 530 nm using a UV–vis spectrophotometer (M2e; Molecular Devices, Sunnyvale, CA, USA). TBARS were calculated using the following equation: TBARS (mg MDA/kg sample) = (sample absorbance − blank absorbance) × 5.2.

### 2.7. pH and WHC

WHC of thigh meat samples was measured at 1, 3, 5, and 7 days at 4 °C using the filter paper press method described by Hofspnn et al. [18], as follows: 0.3 g of thigh meat was placed on filter paper and covered with a plexiglass plate, followed by the application of pressure for 5 min. After measuring the sample area and the total liquid area using a planimeter (Super PLANIXa; Tamaya Technics, Inc., Tokyo, Japan), the WHC index (%) was calculated as meat area/liquid area × 100.

### 2.8. Statistical Analysis

All data were analyzed using a one-way ANOVA (SPSS 18.0; SPSS, Chicago, IL, USA) [19]. Results are indicated as the mean and standard error of the mean (SEM). For weight and weight gain, feed intake, and FCR analysis, *n* = 4; for meat qualities such as the water-holding capacity (WHC), pH, thiobarbituric acid reactive substances (TBARS), and the FA composition, *n* = 5. Differences among groups were determined using Duncan’s test [20] and were considered significant at *p*-values equal to or less than 0.05. The correlation between pH and WHC was analyzed using Pearson’s correlation (SPSS Foundation for Statistical Computing).

## 3. Results

### 3.1. Growth Performance

Data on growth performance are presented in Table 4. The performance of broilers in all experimental treatments showed no difference from that of the control group, except with the SP treatment. At the end of the experiment, broilers fed SP showed significantly higher final BW and BWG than those in the other experimental treatments but FI and FCR values were not different within treatments.

### 3.2. Carcass Characteristics

Carcass trait data are shown in Table 5. The dressed carcass percentage was not influenced by any of the dietary treatments and abdominal fat was not different in broilers fed SP compared to the control. However, abdominal fat deposition was significantly lower in SP-supplemented broilers than in birds treated with SO or FO.

### 3.3. Proximate Composition of Thigh Meat

The effects of ω-3 FAs on the composition of thigh meat are shown in Table 6. There was no influence of any of the treatments on the moisture, crude protein, crude fat, or crude ash content of thigh meat.

### 3.4. Fatty Acid Composition of Thigh Meat

Table 7 shows the FA composition of the thigh meat of broilers fed different dietary lipid sources. Supplementation with lipid sources resulted in significant effects on the FA composition of thigh meat, with the exception of eicosenoic acid (C20:1n9). The three most abundant fatty acids in the chicken thigh meat were oleic acid (35.03–36.13%), palmitic acid (23.21–26.50%), and linoleic acid (14.21–15.56%). Supplementation with SP, which contains high concentrations of palmitic acid and DHA (C22:6n3) (63.44 and 29.40%, respectively), led to a significant increase in the palmitic acid and DHA content of the thigh meat. Supplementation with SO, which is rich in oleic acid, LA, palmitic acid, EPA (C20:5n-3), and DHA (38.41, 17.33, 15.27, 3.14, and 3.70%, respectively), resulted in a major increase in EPA and DHA. Supplementation with FO, which is rich in α-linolenic acid (ALA, C18:3n-3), resulted in a major increase in ALA. The value of ω-6/ω-3 ratio from chicken meat supplemented with SP and SO was about 68% lower than that of broilers in the control group and the ratio in the FO treatment was the lowest (1.86%).

### 3.5. pH, WHC, and TBARS of Broilers Thigh Meat

Table 8 shows the changes in meat quality traits such as TBARS, WHC, and pH during the storage period. As expected, TBARS values in all the dietary treatments increased over the entire storage period. However, broilers fed SP had significantly lower TBARS values in the thigh meat at 7 d than those of the SO and FO treatments. The value of WHC was not affected by the treatment and storage period. Compared to the control group, pH values were not affected by dietary treatments, except for a significant increase in the FO treatment at 1 and 5 days and in the SP and SO treatments at 3 days.

## 4. Discussion

### 4.1. Growth Performance

Shang et al. [21] reported that the addition of omega-3 fats and oils in limited amounts resulted in an improvement of the efficiency of feed and energy utilization, leading to the enhancement of the growth performance of broilers.

There were significantly higher BWGs in the SP treatment than in the other groups, but no difference was found in FI and FCR. These results are inconsistent with the results of Yan and Kim [22], who found that the inclusion of *schizochytrium* JB5 did not affect BWG, FI, or FCR. Rymer et al. [23] also suggested that supplementation with *schizochytrium* did not affect the productive performance of broiler chickens. However, it is somewhat difficult to compare the effect of *schizochytrium* used in the present study on performance with other experiments because the maximum concentrations of SP in other experiments were just 2 g and 7.5 g per kg of diet.

Chin et al. [24] stated that ω-3 FA-rich fish oil has also been reported to decrease the catabolic response caused by immune stimulation and is probably effective in improving growth. However, no effect of SO was observed on the productive performance in the current study. FO treatment also showed no effect on performance, consistent with the study of Olomu and Baracos [25], which reported that no effect of 4.5% flaxseed oil was found on the BWG, FI, and FCR of broilers compared with the control group.

Generally, it has been reported that a dietary addition of a PUFA-rich ingredient has a potential for improving the final BW, BWG, and FCR of poultry [26]. The studies of Crespo and Esteve Garcia [27], Newman et al. [28], and Ferrini et al. [29] suggested the more the unsaturation, the higher digestibility of fat; in other words, the feed efficiency of fat could indicate the degree of unsaturation. This is consistent with the results of Huo et al. [30] and Lopez-Ferrer et al. [31] showing improvement of growth performance with increasing amounts of UFA. However, in the SO and FO treatments containing a higher content of unsaturated fat in the feed during the entire period (Table 3), there were no effects of unsaturated fat levels on the improvement of the BWG, FI, and FCR of broilers in the present study.

### 4.2. Carcass Characteristics

Regardless of the amount of SP used in other experiments, the results were the same as the results we obtained. Yan and Kim [22] reported that the inclusion of the *schizochytrium* JB5 powder did not affect the relative weight of abdominal fat. However, abdominal fat deposition was lower in SP-supplemented broilers than in broilers treated with SO and FO (*p* < 0.01). These results could be explained by the ample DHA of SP that can effectively prevent abdominal fat accumulation [32]. Therefore, even though SP has abundant SFA comparing with SO and FO (Table 1), in the current study, the abdominal fat significantly decreased in broilers fed SP.

### 4.3. Fatty Acid Composition of Thigh Meat

Among the ω-3 FAs, ALA increased the most in the FO treatment, EPA increased the most in the SO treatment, and DHA increased the most in the SP treatment in thigh meat, reflecting the FA composition of the lipid source diets. Broilers fed SP tend to show lower SFA and higher PUFA concentrations in thigh meat than those of the control group, but no significant differences were observed. Inconsistent with the present study, Ribeiro et al. [6] indicated that broilers fed SP had significantly higher concentrations of SFA and a lower concentration of PUFA than those of the control group in thigh meat. However, except for the SP treatment, SO and FO treatments showed significantly lower SFA and higher PUFA concentrations in thigh meat than those of the control group.

Similar to the findings of the present study, Hulan et al. [33] suggested that the ω-3 FAs in poultry meat could be enhanced by increasing the levels of ω-3 FAs in poultry diets and Shin et al. [34] demonstrated that feeding broiler chicken flaxseed oil or fish oil may have resulted in a reduction of ω-6 FAs and an increase of the content of ω-3 FAs in chicken meat. Because the conversion of ω-3 and ω-6 FAs shares the same type of enzymes, a competition occurs between the ω-3 and ω-6 FAs families for the metabolism showing one considerably reduced due to an excess of the other [35].

Huang et al. [36] and Shin et al. [34] reported that chickens fed a fish-meal- or fish-oil-rich diet had substantial contents of ω-3 FAs (EPA and DHA) in the meat. Moreover, Olomu and Baracos [25] suggested that supplementation of ALA-rich flaxseed oil led to an increase in the conversion rate of ALA to EPA and DHA (desaturation and elongation products) in chicken meat. However, ALA, the FAs in the form of predesaturase and pre-elongase activity, was more accumulated in the thigh meat than in the form of EPA and DHA in this study. By seeing the ω-3 contents of chicken meat in Table 7, we can agree with Rymer et al. [23], who reported that broilers accumulated more long-chain ω-3 PUFA (EPA and DHA) in thigh meat when fed a DHA-enriched diet than when supplemented with ALA (the shorter-chain precursor)-enriched diet.

The UK Department of Health recommends the PUFA/SFA ratio in human diets to be <0.45 and the ω-6/ω-3 ratio to be >4.0 [37]. All treatments met the recommended PUFA/SFA ratio levels, and only the FO treatment met the recommended ω-6/ω-3 ratio level. However, the ω-6/ω-3 ratio of the SP and SO treatments also improved and approached the recommended level of 4.0.

### 4.4. pH, WHC, and TBARS of Broilers Thigh Meat

The pH of fresh chicken meat is approximately 5.3–6.5 post slaughter [38] and pH values from all treatments were within this range, which confirms that 20 g addition of ω-3 sources did not affect the freshness of meat, except in the SO treatment at 7 days (6.55). It is generally accepted that pH is one of the main factors affecting WHC [39]. The higher the pH value which is above the meat isoelectric point (approximately pH 5.2), the higher the WHC. Consistent with our results, Qlao et al. [40] reported that broilers muscle with pH values closer to the isoelectric point had higher WHC. These results show that meat quality is also influenced by other factors such as microbial growth, self-extinguishing decomposition ability, and ripening degree [41].

MDA, ketones, and oxidation products are measured by TBARS assay, and the value of TBARS represents the lipid oxidation level. Perceptible rancidity is detected when TBARS values are above 0.8 mg MDA/kg [42]. Increased TBARS values for all the dietary treatments over the entire storage period showed a susceptibility of the meat to oxidation, which was increased with the use of lipid sources rich in polyunsaturated fats [43], but values were within the edible range (0.46 mg/kg). Similarly, another study showed the more storage time, the higher TBARS values of chicken thigh meat that were fed polyunsaturated fat sources and showed that the increase of PUFAs in raw meat produces a significant linear increase of the lipid oxidation in cooked meat [43]. As the content of PUFA increases, lipid oxidation also linearly increases, and the oxidative stability of unsaturated fatty acids (UFA) decreases as the level of unsaturation increases. A decreased oxidative stability of the meat from ω-3-supplemented treatments probably resulted from the abundance of meat with ω-3 long-chain-PUFAs, especially DHA, which are highly susceptible to oxidation. [6,43]. PUFA (such as EPA and DHA) may prevent various lifestyle diseases and promote consumer health. However, an increase in PUFA content affects lipid oxidation and influences flavor, color, and oxidative stability during storage. Moreover, lower TBARS values were shown at 7 d in the thigh meat of broilers fed SP than those of the broilers fed SO and FO. Other studies have shown an interaction between dietary fat source and supplementation with other antioxidants [43]. The lower lipid oxidation of meat at 7 d in the SP treatment than in the SO and FO treatments is probably attributable to the SP containing canthaxanthin and a low amount of UFA.

## 5. Conclusions

In conclusion, we demonstrated that all the dietary omega-3 fatty acid sources could improve the fatty acid composition of chicken meat without negative effects in growth performance, carcass characteristics, and meat quality (pH, water holding capacity). Increased levels of docosahexaenoic acid were found in the thigh meat of broilers fed the *schizochytrium* powder, while eicosapentaenoic acid and docosahexaenoic acid were on the rise in the meat of broilers fed salmon oil, and α-linolenic acid was increased in the meat of broilers fed flaxseed oil. The omega-6/omega-3 ratio of the thigh meat improved in all treatments, approaching the guideline levels. The only negative aspect was a high level of thiobarbituric acid reactive substances, which was, however, within the edible range. Our results indicate the possibility to supplement broiler diets with *schizochytrium* powder, salmon oil, and flaxseed oil as omega-3 fatty acid sources to produce meat with a good nutritional quality at a 2% dietary supplement level. Very significant increases in the omega-3 fatty acids (eicosapentaenoic acid and docosahexaenoic acid) were observed in chicken thigh meat. These translated into increases in eicosapentaenoic acid and docosahexaenoic acid in the meat, which would allow consumers to easily enhance their intake of these important nutrients for health benefits.

## Figures and Tables

**Table 1:** Ingredients and nutrient level of experimental diets (as-fed basis).

Items	Starter Diet (0 to 7 d)	Grower Diet (8 to 21 d)	Finisher Diet (22 to 35 d)
Ingredients, %			
Corn, US no. 3	51.35	56.06	58.15
Soybean meal-45% CP	37.45	32.55	30.43
Wheat	4.00	4.00	4.00
Soybean oil	3.07	3.57	4.03
Mono dicalcium phosphate	1.35	1.18	0.95
Limestone	1.60	1.58	1.57
Sodium chloride	0.25	0.25	0.25
Methionine-99%	0.33	0.25	0.22
Lysine-78%	0.20	0.16	0.00
Vitamin and mineral premix ^1^	0.20	0.20	0.20
Sodium bicarbonate	0.20	0.20	0.20
Total	100.00	100.00	100.00
Nutrient level ^2^			
ME_n_, kcal/kg	3025	3100	3150
Crude protein, %	22.00	20.00	19.00
Crude fiber	4.76	4.54	6.19
Ca, %	0.95	0.90	0.85
Nonphytate P, %	0.45	0.	0.35
Lys, %	1.42	1.25	1.10
Met + Cys, %	1.05	0.92	0.87

^1^ Provided per kilogram of the complete diet: vitamin A (from vitamin A acetate), 12,500 IU; vitamin D_3_, 2500 IU; vitamin E (from DL-α-tocopheryl acetate), 20 IU; vitamin K_3_, 2 mg; vitamin B_1_, 2 mg; vitamin B_2_, 5 mg; vitamin B_6_, 3 mg; vitamin B_12_, 18 µg; calcium pantothenate, 8 mg; folic acid, 1 mg; biotin, 50 µg; niacin, 24 mg. Fe (as FeSO_4_·7H_2_O), 40 mg; Cu (as CuSO_4_·H_2_O), 8 mg; Zn (as ZnSO_4_·H_2_O), 60 mg; Mn (as MnSO_4_·H_2_O) 90 mg; Mg (MgO) as 1500 mg. ^2^ Measured value.

**Table 2:** Fatty acids contents (% of total fatty acids) of microalgae, salmon oil, and flaxseed oil.

Items	Treatments		
	Microalgae Powder	Salmon Oil	Flaxseed Oil
Myristic acid (C14:0)	4.14	3.10	0.06
Palmitic acid (C16:0)	63.44	15.27	6.62
Palmitoleic acid (C16:1n7)	0.05	3.20	0.07
Stearic acid (C18:0)	1.55	4.43	4.34
Oleic acid (C18:1n9)	0.02	38.41	17.13
Vaccenic acid (C18:1n7)	0.09	4.23	1.45
Linoleic acid (C18:2n6)	0.00	17.33	13.91
α-Linolenic acid (C18:3n3)	0.07	6.44	54.93
γ-Linolenic acid (C18:3n6)	0.08	0.28	0.37
Eicosenoic acid (C20:1n9)	0.00	0.00	0.96
Arachidonic acid (C20:4n6)	0.16	0.38	0.09
Eicosapentaenoic acid (C20:5n3)	0.92	3.14	0.06
Adrenic acid (C22:4n6)	0.07	0.09	0.00
Docosahexaenoic acid (C22:6n3)	29.40	3.70	0.00
Total	100.00	100.00	100.00
Saturated fatty acids (SFA)	69.13	22.80	11.03
Unsaturated fatty acids (UFA)	30.87	77.20	88.97
Monounsaturated fatty acid (MUFA)	0.16	45.85	19.61
Poly unsaturated fatty acid (PUFA)	30.70	31.36	69.36
MUFA/SFA	0.00	2.01	1.78
PUFA/SFA	0.44	1.38	6.29
ω-6/ω-3	0.01	1.36	0.26

**Table 3:** Fatty acids contents (% of total fatty acids) of starter *, grower **, and finisher *** diets.

Items	Treatments ^1^											
	CON			SP			SO			FO		
	1 *	2 **	3 ***	1 *	2 **	3 ***	1 *	2 **	3 ***	1 *	2 **	3 ***
C14:0	1.93	2.04	1.95	2.04	2.01	1.88	1.96	2.20	1.92	1.60	1.67	1.59
C16:0	24.54	25.49	24.56	27.25	25.98	25.61	22.77	22.81	23.02	21.28	21.82	21.29
C16:1n7	1.83	1.94	1.86	1.87	2.01	1.79	2.11	2.23	1.98	1.63	1.69	1.54
C18:0	10.06	11.16	10.54	8.88	9.18	9.24	8.43	9.89	9.05	9.04	9.78	9.22
C18:1n9	27.68	28.82	29.86	25.24	27.37	28.72	28.50	31.34	30.86	27.13	27.28	28.15
C18:1n7	2.02	1.90	2.80	2.38	2.18	2.59	2.53	2.47	2.69	1.52	2.10	2.01
C18:2n6	25.64	22.34	23.62	25.74	25.62	24.79	27.18	21.29	24.44	23.77	22.06	22.32
C18:3n3	0.00	0.02	0.00	0.00	0.00	0.00	0.00	0.07	0.00	0.00	0.09	0.00
C18:3n6	2.06	1.84	0.95	2.12	2.28	1.61	3.20	2.77	2.26	11.18	10.34	10.78
C20:1n9	2.82	3.66	3.14	1.72	0.91	1.92	0.94	3.08	2.22	1.72	2.53	2.37
C20:4n6	0.07	0.10	0.10	0.05	0.05	0.14	0.14	0.08	0.17	0.07	0.07	0.08
C20:5n3	0.27	0.19	0.11	0.34	0.23	0.18	0.72	0.65	0.54	0.24	0.13	0.16
C22:4n6	0.18	0.10	0.12	0.15	0.06	0.05	0.21	0.13	0.00	0.16	0.09	0.07
C20:6n3	0.88	0.40	0.39	2.22	2.12	1.46	1.31	0.99	0.86	0.66	0.35	0.42
Total	100.00	100.00	100.00	100.00	100.00	100.00	100.00	100.00	100.00	100.00	100.00	100.00
SFA	36.54	38.69	37.06	38.17	37.17	36.74	33.16	34.89	33.99	31.92	33.27	32.10
UFA	63.46	61.31	62.94	61.83	62.83	63.26	66.84	65.11	66.01	68.08	66.73	67.90
MUFA	34.36	36.32	37.67	31.22	32.47	35.02	34.08	39.11	37.74	32.00	33.61	34.07
PUFA	29.11	24.99	25.27	30.61	30.37	28.24	32.76	25.99	28.27	36.07	33.12	33.83
MUFA/SFA	0.94	0.94	1.02	0.82	0.87	0.95	1.03	1.12	1.11	1.00	1.01	1.06
PUFA/SFA	0.80	0.65	0.68	0.80	0.82	0.77	0.99	0.75	0.83	1.13	1.00	1.05
ω-6/ω-3	8.09	9.31	16.62	5.54	5.56	7.72	5.26	4.89	6.72	1.99	2.06	1.98

^1^ CON = basal diet; SP = basal diet + 20 g/kg *Schizochytrium*; SO = basal diet + 20 g/kg salmon oil; FO = basal diet + 20 g/kg flaxseed oil.

**Table 4:** Effects of dietary microalgae, salmon oil, and flaxseed oil on growth performance of broiler chickens ^1^.

Items	Dietary Treatments ^2^				SEM ^3^	*p*-Value
	CON	SP	SO	FO		
Initial BW ^4^, g	39.3	39.4	40.0	39.6	0.17	0.50
Final BW, g	2608.6 ^b^	2673.0 ^a^	2578.8 ^b^	2582.1 ^b^	12.38	<0.01
BW gain, g	2569.2 ^b^	2633.6 ^a^	2538.9 ^b^	2542.5 ^b^	12.42	<0.01
Feed intake, g	3630.3	3754.1	3635.3	3626.2	20.35	0.08
FCR	1.4	1.4	1.4	1.4	0.01	0.79

^a,b^ Means in the same row with different superscripts differ significantly (*p* < 0.05). *n* = 4. ^1^ Data are least square means of 4 replicates per treatment. ^2^ CON = basal diet; SP = basal diet + 20 g/kg *Schizochytrium*; SO = basal diet + 20 g/kg salmon oil; FO = basal diet + 20 g/kg flaxseed oil. ^3^ Standard error of means. ^4^ BW = body weight; FCR = feed conversion ratio. No mortality was observed during the experimental periods

**Table 5:** Effects of dietary Schizochytrium, salmon oil, and flaxseed oil on carcass characteristics in broiler chickens ^1^.

Items (%)	Dietary Treatments ^2^				SEM ^3^	*p*-Value
	CON	SP	SO	FO		
Carcass	71.0	71.6	72.8	73.1	0.33	0.06
Abdominal fat	1.0 ^bc^	0.7 ^c^	1.5 ^a^	1.4 ^ab^	0.10	<0.01

^a–c^ Means in the same of row with different superscript differ significantly (*p* < 0.05). ^1^ Data are least square means of 4 replicates per treatment. ^2^ CON = basal diet; SP = basal diet + 20 g/kg *Schizochytrium*; SO = basal diet + 20 g/kg salmon oil; FO = basal diet + 20 g/kg flaxseed oil. ^3^ Standard error of means.

**Table 6:** Effects of dietary microalgae, salmon oil, and flaxseed oil on proximate composition of thigh meat in broiler chickens ^1^.

Items (%)	Dietary Treatments ^2^				SEM ^3^	*p*-Value
	CON	SP	SO	FO		
Dry matter	25.11	24.64	24.69	24.19	0.017	0.34
Crude protein	18.97	19.23	19.62	19.00	0.010	0.74
Crude fat	3.29	3.35	3.42	3.50	0.007	0.77
Crude ash	0.82	0.88	0.81	0.88	0.006	0.30

*n* = 5. ^1^ Data are least square means of 4 replicates per treatment. ^2^ CON = basal diet; SP = basal diet + 20 g/kg *Schizochytrium*; SO = basal diet + 20 g/kg salmon oil; FO = basal diet + 20 g/kg flaxseed oil. ^3^ Standard error of means.

**Table 7:** Effects of dietary microalgae, salmon oil, and flaxseed oil on fatty acids contents (% of total fatty acids) of thigh meat in broiler chickens ^1^.

Items	Treatments ^2^				SEM ^3^	*p*-Value
	CON	SP	SO	FO		
C14:0	1.49 ^a^	1.49 ^a^	1.53 ^a^	1.22 ^b^	0.031	<0.001
C16:0	24.93 ^b^	26.50 ^a^	24.32 ^c^	23.21 ^d^	0.088	<0.001
C16:1n7	3.74 ^bc^	4.69 ^a^	3.58 ^c^	4.12 ^b^	0.139	<0.001
C18:0	8.81 ^a^	6.89 ^c^	8.17 ^b^	7.07 ^c^	0.141	<0.001
C18:1n9	35.73 ^a^	35.70 ^ab^	36.13 ^a^	35.03 ^b^	0.215	0.045
C18:1n7	4.22 ^a^	4.02 ^a^	3.91 ^ab^	3.55 ^b^	0.124	0.032
C18:2n6	15.56 ^a^	14.77 ^c^	15.19 ^b^	14.21 ^d^	0.086	<0.001
C18:3n3	0.69 ^d^	1.13 ^c^	1.50 ^b^	7.37 ^a^	0.107	<0.001
C18:3n6	0.16 ^a^	0.00 ^b^	0.00 ^b^	0.00 ^b^	0.000	<0.001
C20:1n9	2.58	0.40	2.63	2.54	0.148	0.214
C20:4n6	1.51 ^a^	0.74 ^c^	1.24 ^b^	0.79 ^c^	0.052	<0.001
C20:5n3	0.00 ^c^	0.40 ^b^	0.54 ^a^	0.38 ^b^	0.040	<0.001
C22:4n6	0.24 ^a^	0.11 ^c^	0.16 ^b^	0.12 ^c^	0.008	<0.001
C20:6n3	0.34 ^c^	1.40 ^a^	1.09 ^b^	0.40 ^c^	0.024	<0.001
Total	100.00	100.00	100.00	100.00		
SFA	35.23 ^a^	34.87 ^a^	34.02 ^b^	31.50 ^c^	0.189	<0.001
UFA	64.77 ^c^	65.13 ^c^	65.98 ^b^	68.50 ^a^	0.189	<0.001
MUFA	46.27 ^a^	46.59 ^a^	46.24 ^a^	45.24 ^b^	0.255	0.03
PUFA	18.50 ^c^	18.54 ^c^	19.74 ^b^	23.27 ^a^	0.149	<0.001
MUFA/SFA	1.31 ^b^	1.34 ^b^	1.36 ^b^	1.44 ^a^	0.015	<0.001
PUFA/SFA	0.53 ^c^	0.53 ^c^	0.58 ^b^	0.74 ^a^	0.005	<0.001
ω-6/ω-3	17.04 ^a^	5.38 ^b^	5.29 ^b^	1.86 ^c^	0.544	<0.001

^a–d^ Means in the same row with different superscripts differ significantly (*p* < 0.05). *n* = 5. ^1^ Data are least square means of 3 replicates per treatment. ^2^ CON = basal diet; SP = basal diet + 20 g/kg *Schizochytrium*; SO = basal diet + 20 g/kg salmon oil; FO = basal diet + 20 g/kg flaxseed oil. ^3^ Standard error of means.

**Table 8:** Effects of dietary microalgae, salmon oil, and flaxseed oil on thiobarbituric acid reactive substances (TBARS), Water holding capacity (WHC), and pH of thigh meat in broiler chickens ^1^.

Items	Days	Treatments ^2^				SEM ^3^	*p*-Value
		CON	SP	SO	FO		
TBARS (mg MDA/kg meat)	1	0.041 ^Db^	0.055 ^Ca^	0.052 ^Ca^	0.057 ^Ca^	0.002	<0.001
	3	0.052 ^C^	0.053 ^C^	0.055 ^C^	0.052 ^C^	0.001	0.703
	5	0.060 ^Bc^	0.077 ^Bb^	0.086 ^Bb^	0.098 ^Ba^	0.004	<0.001
	7	0.065 ^Ac^	0.152 ^Ab^	0.160 ^Aa^	0.163 ^Aa^	0.011	<0.001
	**SEM ^3^**	0.002	0.011	0.011	0.012		
	***p*-value**	<0.001	<0.001	<0.001	<0.001		
WHC (%)	1	44.66	45.76	51.73	44.54	1.203	0.094
	3	42.56	44.47	49.78	43.50	1.243	0.163
	5	38.53	43.36	46.45	40.81	1.272	0.141
	7	36.92	37.99	43.38	38.33	1.051	0.124
	**SEM ^3^**	1.024	1.492	1.224	1.058		
	***p*-value**	0.009	0.286	0.058	0.149		
pH	1	6.19 ^b^	6.19 ^b^	6.22 ^C^^b^	6.27 ^D^^a^	0.009	<0.001
	3	6.30 ^b^	6.37 ^a^	6.35 ^B^^a^	6.31 ^C^^b^	0.008	<0.001
	5	6.35 ^a^	6.34 ^a^	6.33 ^B^^a^	6.38 ^B^^b^	0.005	<0.05
	7	6.33	6.46	6.55 ^A^	6.49 ^A^	0.060	0.675
	**SEM ^3^**	0.059	0.059	0.031	0.021		
	***p*-value**	0.815	0.768	<0.001	<0.001		

^a–c^ Means in the same row with different superscripts differ significantly (*p* < 0.05). ^A–D^ Means in the same column with different superscripts differ significantly (*p* < 0.05). *n* = 5. ^1^ Data are least square means of 3 replicates per treatment. ^2^ CON = basal diet; SP = basal diet + 20 g/kg *Schizochytrium*; SO = basal diet + 20 g/kg salmon oil; FO = basal diet + 20 g/kg flaxseed oil. ^3^ Standard error of means.

## Data Availability

Not applicable.

## References

[1] Candela C.G., Lopez L.B., Kohen V.L. (2011). Importance of a balanced omega 6/omega 3 ratio for the mainte nance of health. Nutritional recommendations. Nutr. Hosp..

[2] Simopoulos A.P. (2002). The importance of the ratio of omega-6/omega-3 essential fatty acids. Biomed. Pharmacother..

[3] Simopoulos A.P. (2014). An increase in the omega-6/omega-3 fatty acid ratio increases the risk for obesity. Nutrients.

[4] Health and Welfare Canada (1990). Nutrition Recommendations: The Report of the Scientific Review Committee.

[5] Lopez-Ferrer S., Baucells M.D., Barroeta A.C., Grashorn S.P. (2001). n-3 enrichment of chicken meat. 1. Use of very long-chain fatty acids in chicken diets and their influence on meat quality: Fish oil. Poult. Sci..

[6] Ribeiro T., Lordelo M.M., Alves S.P., Bessa R.J.B., Costa P., Lemos J.P.C., Ferreira L.M.A., Fontes C.M.G.A., Prates J.A.M. (2013). Direct supplementation of diet is the most efficient way of enriching broiler meat with n-3 long-chain polyunsaturated fatty acids. Br. Poult. Sci..

[7] Gonzalez-esquerra R., Leeson S. (2001). Alternatives for enrichment of eggs and chicken meat with omega-3 fatty acids. Can. J. Anim. Sci..

[8] Trentacoste E.M., Shrestha R.P., Smith S.R., Gle C., Hartspnn A.C., Hildebrand M., Gerwick W.H. (2013). Metabolic engi neering of lipid catabolism increases microalgal lipid accumulation without compromising growth. Proc. Natl. Acad. Sci. USA.

[9] Park J.H., Upadhaya S.D., Kim I.H. (2015). Effect of dietary Marine microalgae (Schizochytrium) powder on egg production, blood lipid profiles, egg quality, and fatty acid composition of egg yolk in layers. Asian-Australas. J. Anim. Sci..

[10] Baldwin N. (1997). Application for Approval of DHA-Rich Oil.

[11] Barclay W.R., Meager K.M., Abril J.R. (1994). Heterotrophic production of long chain omega-3 fatty acids utilizing algae and algae-like microorganisms. J. Appl. Phycol..

[12] Raghukumar S. (2008). Thraustochytrid marine protists: Production of PUFAs and other merging technologies. Mar. Biotechnol..

[13] National Research Council (1994). Nutrient Requirements of Poultry.

[14] AOAC (Association of Official Analytical Chemists) (1990). Official Methods of Analysis of AOAC International.

[15] Folch J., Lees M., Sloane Stanley G.H. (1957). A simple method for the isolation and purification of total lipids from animal tissues. J. Biol. Chem..

[16] Morrison W.R., Smith L.M. (1964). Identification of ceramide monohexoside and ceramide dihexoside in bovine milk. Biochim. Biophys. Acta. Spec. Sect. Lipids Relat. Subj..

[17] Witte V.C., Krause G.F., Bailey M.E. (1970). A new extraction method for determining 2-thiobarbituric acid values of pork and beef during storage. J. Food Sci..

[18] Hofspnn K., Hamm R., Bluchel E.M. (1982). Neunes Uber die bestimmung der wasserbindung des fleisches mithilfe der filterpapier press methode. Fleischwiry.

[19] IBM SPSS Statistics for Windows (2017). SPSS Statistics for Windows.

[20] Duncan B.D. (1955). Multiple Range and F-test. Biometrics.

[21] Shang X.G., Wang F.L., Li D.F., Yin J.D., Li J.Y. (2004). Effects of dietary conjugated linoleic acid on the productivity of laying hens and egg quality during refrigerated storage. Poult. Sci..

[22] Yan L., Kim I.H. (2013). Effects of dietary ω-3 fatty acid-enriched microalgae supplementation on growth performance, blood profiles, meat quality, and fatty acid composition of meat in broilers. J. Appl. Anim. Res..

[23] Rymer C., Gibbs R.A., Givens D.I. (2010). Comparison of algal and fish sources on theoxidative stability of poultry meat and its enrichment with omega-3 polyunsaturated fatty acids. Poult. Sci..

[24] Chin S.F., Storkson G.M., Albright K.J., Cook M.E., Pariza M.W. (1992). Conjugated linoleic acid is a growth factor of rats as shown by enhanced weight gain and improved feed efficiency. J. Nutr..

[25] Olomu J.M., Baracos V.E. (1991). Influence of dietary flaxseed oil on the performance, muscle protein deposition, and fatty acid composition of broiler chicks. Poult. Sci..

[26] Alagawany M., Elnesr S.S., Farag M.R., El-Hack A., Mohamed E., Khafaga A.F., Dhama K. (2019). Omega-3 and Omega-6 fatty acids in poultry nutrition: Effect on production performance and health. Animals.

[27] Crespo N., Esteve-Garcia E. (2002). Dietary polyunsaturated fatty acids decrease fat deposition in separable fat depots but not in the remainder carcass. Poult. Sci..

[28] Newman R.E., Bryden W.L., Fleck E., Ashes J.R., Buttemer W.A., Storlien L.H., Downing J.A. (2002). Dietary n-3 and n-6 fatty acids alter avian metabolism: Metabolism and abdominal fat deposition. Br. J. Nutr..

[29] Ferrini G., Baucells M.D., Esteve-Garcia E., Barroeta A.C. (2008). Dietary polyunsaturated fat reduces skin fat as well as abdominal fat in broiler chickens. Poult. Sci..

[30] Huo W., Li M., Wang J., Wang Z., Huang Y., Chen W. (2019). On growth performance, nutrient digestibility, blood T lymphocyte subsets, and cardiac antioxidant status of broilers. Anim. Nutr..

[31] Lopez-Ferrer S., Baucells M.D., Barroeta A.C., Grashorn M.A. (2001). n-3 enrichment of chicken meat. 2. Use of precursors of long-chain polyunsaturated fatty acids: Linseed Oil. Poult. Sci..

[32] Yu J., Ma Y., Sun J., Ran L., Li Y., Wang N., Yu T., Gao W., Jia W., Jiang R. (2017). Microalgal oil from Schizochytrium sp. prevents HFD-induced sbdominal fat sccumulation in mice. J. Am. Coll. Nutr..

[33] Hulan H.W., Proudfoot F.G., Ackspn R.G., Ratnayayake W.M.N. (1988). Omega-3 fatty acid levels and performance of broiler chickens fed redfish meal or redfish oil. Can. J. Anim. Sci..

[34] Shin D., Kakani G., Karimi A., Cho Y.M., Kim S.W., Ko Y.G., Park J.H. (2011). Influence of dietary conjugated linoleic acid and its combination with flaxseed oil or fish oil on saturated fatty acid and n-3 to n-6 fatty acid ratio in broiler chicken meat. Asian-Australas. J. Anim. Sci..

[35] Schmitz G., Ecker J. (2008). The opposing effects of n−3 and n−6 fatty acids. Prog. Lipid Res..

[36] Huang Z., Leibovitz H., Lee C.M., Millar R. (1990). Effect of dietary fish oil on. omega-3 fatty acid levels in chicken eggs and thigh flesh. J. Agric. Food Chem..

[37] HMSO (1994). Nutritional Aspects of Cardiovascular Disease.

[38] Hertanto B.S., Nursplasari C.D.A., Nuhriawangsa A.M.P., Ahyadi M.C., Kartikasari L.R. (2018). The physical and microbiological quality of chicken meat in the different type of enterprise poultry slaughterhouse: A case study in Karanganyar District. IOP Conf. Ser. Earth Environ. Sci..

[39] Keenan D.F., Desmond E.M., Hayes J.E., Kenny T.A., Kerry J.P. (2010). The effect of hot-boning and reduced added phosphate on the processing and sensory properties of cured beef prepared from two forequarter muscles. Meat Sci..

[40] Qiao M., Fletcher D.L., Smith D.P., Northcutt J.K. (2001). The effect of broiler breast meat color on pH, moisture, water-holding capacity, and emulsification capacity. Poult. Sci..

[41] Lee S.K., Ju M.K., Kim Y.S., Kang S.M., Choi Y.S. (2005). Quality comparison between Korean native black ground pork and modern genotype ground pork during refrigerated storage. Korean J. Food Sci. Anim. Resour..

[42] O’Neill L.M., Galvin K., Morrissey P.A., Buckley D.J. (1998). Comparison of effects of dietary olive oil, tallow and vitamin E on the quality of broiler meat and meat products. Br. Poult. Sci..

[43] Cortinas L., Barrpeta A., Villaverde C., Galobart J., Guardiola F., Baucells M.D. (2005). Influence of the dietary polyunsaturation level on chicken meat quality: Lipid oxidation. Poult. Sci..

